# First record and redescription of *Macandrewella cochinensis* Gopalakrishnan, 1973 (Copepoda, Scolecitrichidae) from the Red Sea, with notes on swarm formation

**DOI:** 10.3897/zookeys.344.5519

**Published:** 2013-10-22

**Authors:** Mohsen M. El-Sherbiny, Ali M. Al-Aidaroos

**Affiliations:** 1 Department of Marine Biology, King Abdulaziz University, Jeddah 21589, Saudi Arabia; 2 Department of Marine Sciences, Suez Canal University, Ismailia 41522, Egypt

**Keywords:** Zooplankton, copepods, *Macandrewella cochinensis*, swarm, Red Sea

## Abstract

During a study of the epipelagic zooplankton carried out near the fringing reef around Sharm El-Sheikh area, in the northern Red Sea, female and male specimens of the poorly known calanoid copepod *Macandrewella cochinensis* Gopalakrishnan, 1973 were collected. This is the first record of species occurrence in the Red Sea. *Macandrewella cochinensis* was previously known only from the offshore water of Cochin, south west of India. The Red Sea specimens are described in details herein to allow their comparison with the specimens from the type locality, because original description of *M. cochinensis* is incomplete and causes some taxonomic confusion. The most important characters that may have been overlooked in the original description are: shape of projections of the female distolateral prosomal borders, details of morphology of the asymmetrical female genital double-somite and presence of leg 5 in female.

## Introduction

Members of the family Scolecitrichidae are distributed from pelagic to benthopelagic waters of the world oceans. The boundaries of the family Scolecitrichidae are not well defined as reported by Vyshkvartzeva (2001), Ohtsuka et al. (2003) and Boxshall and Halsey (2004). Boxshall and Halsey (2004) have considered this family to contain about 23–26 genera, however, less number of genera have been included in the family by Markhaseva and Ferrari (2005).

The genus *Macandrewella* Scott, 1909 belongs to the family Scolecitrichidae and has so far accommodated 12 nominal species (Razouls et al. 2013). The genus has hitherto been recorded exclusively from tropical and subtropical waters between 30°N and 20°S in the Indo-Pacific waters (Ohtsuka et al. 2002). Most members of the genus are hyperbenthic and have been collected from the near bottom samples on the continental shelves and slopes. Ohtsuka Nishida and Nakaguchi described in 2002 *Macandrewella stygiana* and *Macandrewella omorii* from the southern Japan from the near bottom at depth of 95–467 m. Farran (1936) collected three species (*Macandrewella asymmetrica*, *Macandrewella mera* and *Macandrewella sewelli*) from the Great Barrier Reef when a plankton net accidentally touched the bottom at a depth of 200 m. *Macandrewella cochinensis* described by Gopalakrishnan (1973) was found from 200 m to the surface, *Macandrewella joanae* was sampled by Scott (1909) from 1000 m to the surface. Other species were collected in vertical or surface plankton hauls where no exact depths of collection were specified (Ohtsuka et al. 2002). Ohtsuka et al. (2002) gave an excellent review and a key for all *Macandrewella* species recorded worldwide on the basis of shape of female genital double-somite, presence or absence of a female leg 5, the structure of the second and third exopodal segments of the male right leg 5 and the shape of the right endopod segment of the male leg 5.

Only one species *Macandrewella chelipes* Giesbrecht, 1896 has been recorded from the Red Sea (Giesbrecht 1896, Campaner 1989, El-Sherbiny 1997). During our plankton sampling in the Red Sea, another species of *Macandrewella* was first time found in swarms at surface waters. It is identified as *Macandrewella cochinensis* which up to now has only been recorded from the type locality off Cochin, south coast of India (10°10'N, 75°46'E). This paper describes *Macandrewella cochinensis* collected near a reef in a semi-enclosed small shallow bay in the northern Red Sea. Its habit of swarming in surface waters is also discussed.

## Materials and methods

**Figure 1.**
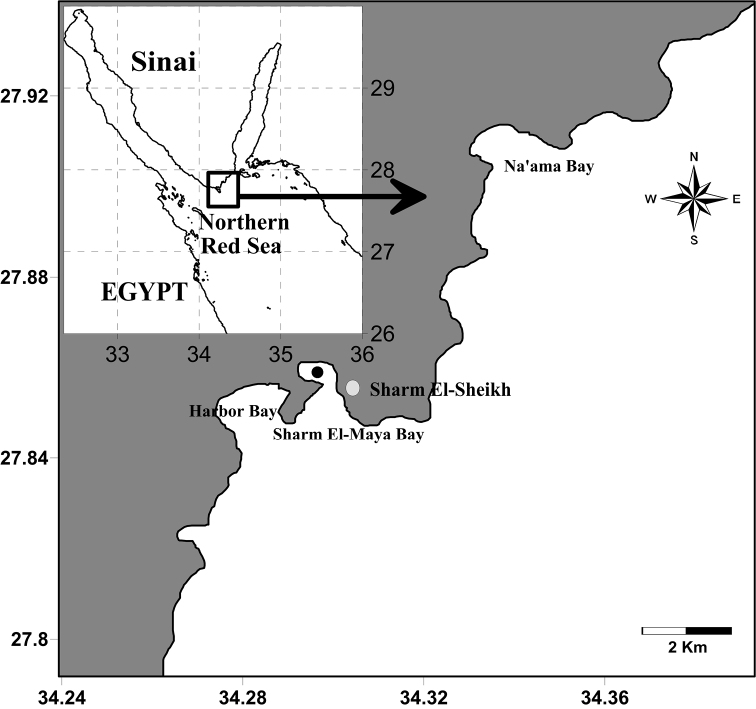
Location of the sampling site (Black circle).

The specimens were collected from the entrance of a semi-enclosed bay called Sharm El-Maya (6–9 m in depth), the northern Red Sea (27°51.234'N, 34°17.605'E, Fig. 1) at 4 PM local time on 5^th^ of December 2011. The plankton samples were collected within 50 m of a fringing reef using a plankton net (diameter 1m, mesh size 0.5 mm). The net was towed for 10 minutes at a speed of about 2 knots. The collected specimens were concentrated and fixed in a 4% neutralized formalin-seawater solution immediately after collection and then transferred in 70% ethanol, sorted and examined using differential interference contrast microscope (Olympus BH-2 and CX41). Drawings were made with the aid of a camera lucida and all measurements were made using an ocular micrometer. The terminology in the description follows Huys and Boxshall (1991). For scanning electronic microscopy (SEM), whole copepods or dissected parts were mounted on stubs, dehydrated with liquid nitrogen, coated with white gold, and examined in a JEOL, JSM-5600LV scanning electron microscope. Specimens are deposited at the Zoological Institute, Russian Academy of Science, Saint Petersburg, Russian Federation (No. 91067) and in the Marine Science Department, Suez Canal University, Egypt. Stages and sexes of individuals comprising the swarm were identified.

## Systematics

### Order Calanoida Sars, 1903
Family Scolecitrichidae Giesbrecht, 1892
*Macandrewella* A. Scott, 1909

#### Macandrewella cochinensis

Gopalakrishnan, 1973

http://species-id.net/wiki/Macandrewella_cochinensis

Figs 2 –7

##### Material examined.

Nine adult females and eight adult males collected from Sharm El-Maya Bay located in the entrance of Sharm El-Sheikh City, the northern Red Sea on 5 December 2011.

Body length. Female: 2.88–3.15 mm (mean±SD=2.99±0.09 mm, *n*=6). Male: 2.83–3.21 mm (2.98±0.13 mm, *n*=6).

##### Female.

**Figure 2.**
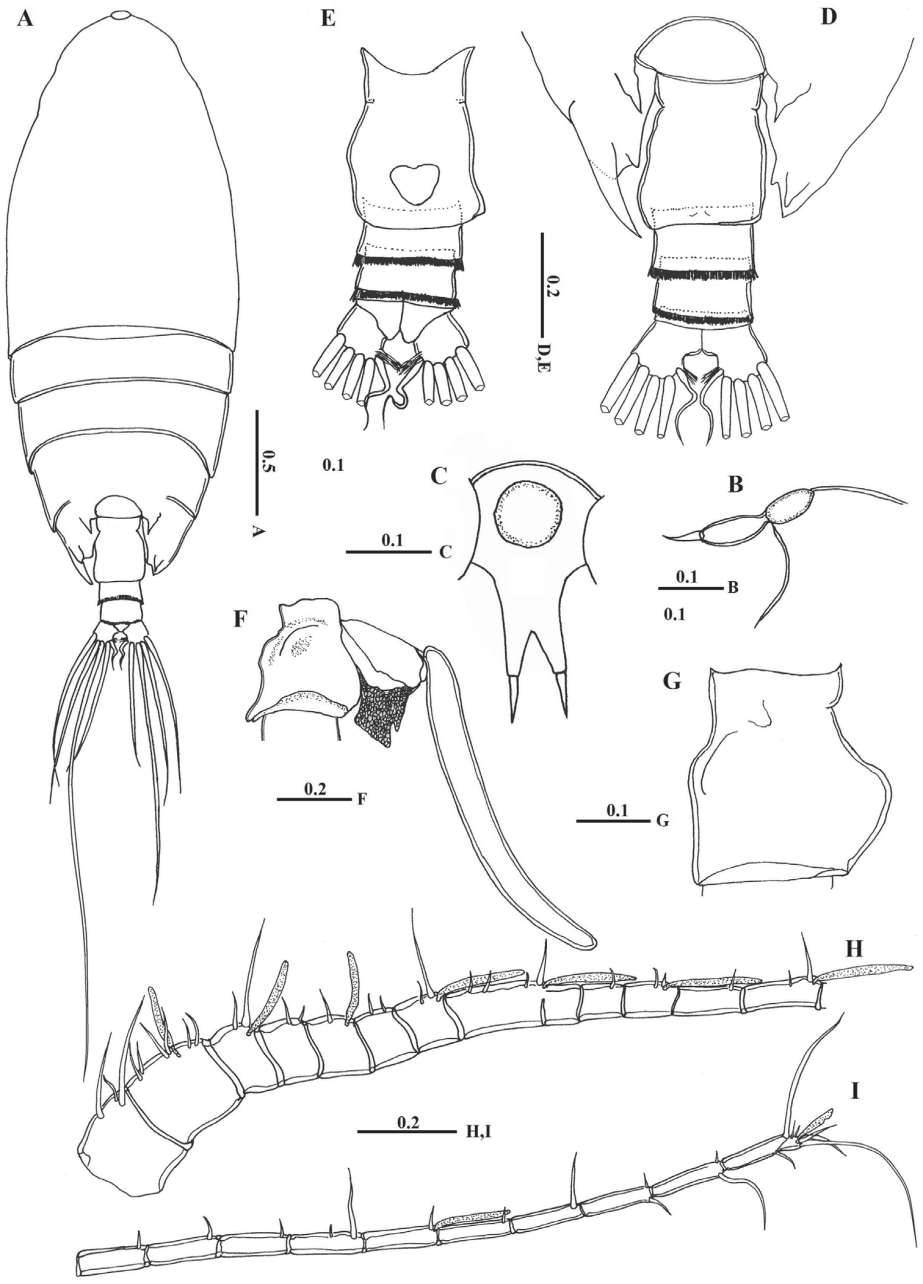
*Macandrewella cochinensis* female from the northern Red Sea. **A** habitus, dorsal view **B** rostrum, lateral view **C** rostrum, ventral view **D** posterior prosome and urosome, dorsal view **E** urosome, ventral view **F** genital double-somite with spermatophore, lateral view (right) **G** genital double-somite, lateral view (right) **H–I** antennules. All scale bars in mm.

**Figure 3.**
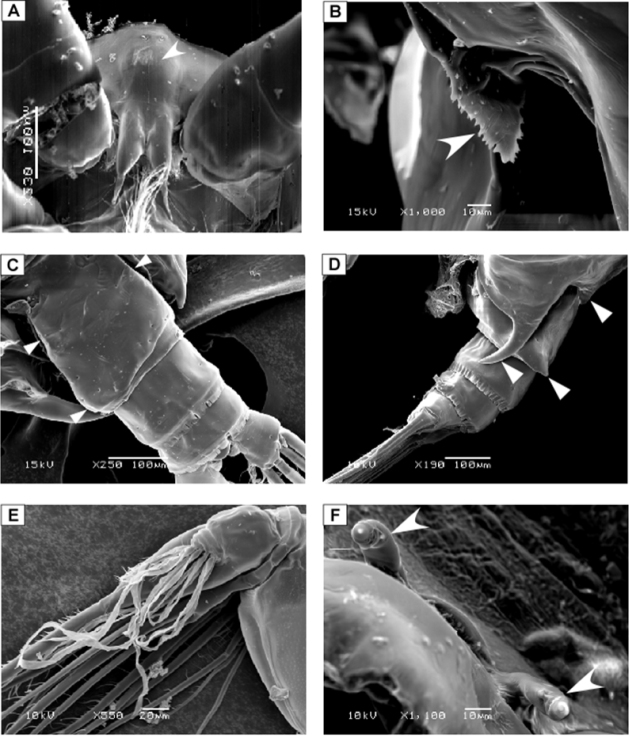
SEM micrographs of *Macandrewella cochinensis* female from the northern Red Sea. **A** rostrum and cuticular lens indicated by arrow, ventral view **B** serration of postero-dorsolateral process of prosomal end indicated by arrow, lateral view **C** urosome, anterodorsal protrusions and posterodorsal swelling on left side indicated by arrows, dorsal view **D** urosome, posterodorsal swelling on left side indicated by arrow, lateral view (left) **E** maxillary endopod **F** leg 5 indicated by arrow.

Body (Fig. 2A) robust; cephalosome completely fused to first pediger, protruding anteroventerally into bifurcated rostrum; rostrum (Figs 2B–C) with pair of slender filaments; single median cuticular lens present at base of rostrum (Figs 2B–C, 3A). Pedigers 4 and 5 partially fused, with incomplete suture visible dorsally and ventrolaterally; posterior margin asymmetrical, left one longer; each with 1 pairs of processes on each side, postero-dorsolateral projecting on each side lamellar with serrated margin, asymmetrical ventrolateral processes curved ventromedially at tip, slightly exceeding the posterior end of genital double somite on left side and slightly exceeding half length of genital double-somite on right (Figs 2D, 3B, D). Urosome (Figs 2E, D) short, approximately one-fifth as long as prosome; of 4 free somites. Genital double-somite asymmetrical with unequal anterodorsal protrusion on each side and posterodorsal swelling on left side (Figs 2F, G, 3C, D); genital area usually with sausage-like spermatophore (Fig. 2F); genital operculum wider than long, located distoventrally (Fig. 2E). Fourth urosomite (anal somite) very short, telescoped into proceeding somite. Caudal rami symmetrical with 5 caudal setae, left middle seta (V) 1.5 times as long as right one.

Antennules (Figs 2H, I) symmetrical, 23-segmented, extending nearly to posterior border of second somite. Segmentation pattern and setal armature elements as follows: I-3, II-IV, 6+ae (II-2, III-2+ae, IV-2), V-2+ae, VI-2, VII-1+ae, VIII-2, IX-2+ae, X-XII-4+ae, XIII-1, XIV--2+ae, XV-1, XVI-2+ae, XVII-1, XVIII-1, XIX-1, XX-2, XXI-1+ae, XXII-1, XXIII-1, XXIV-1+1, XXV-1+1, XXVI-1+1, XXVI-XXVIII-5+ae.

**Figure 4.**
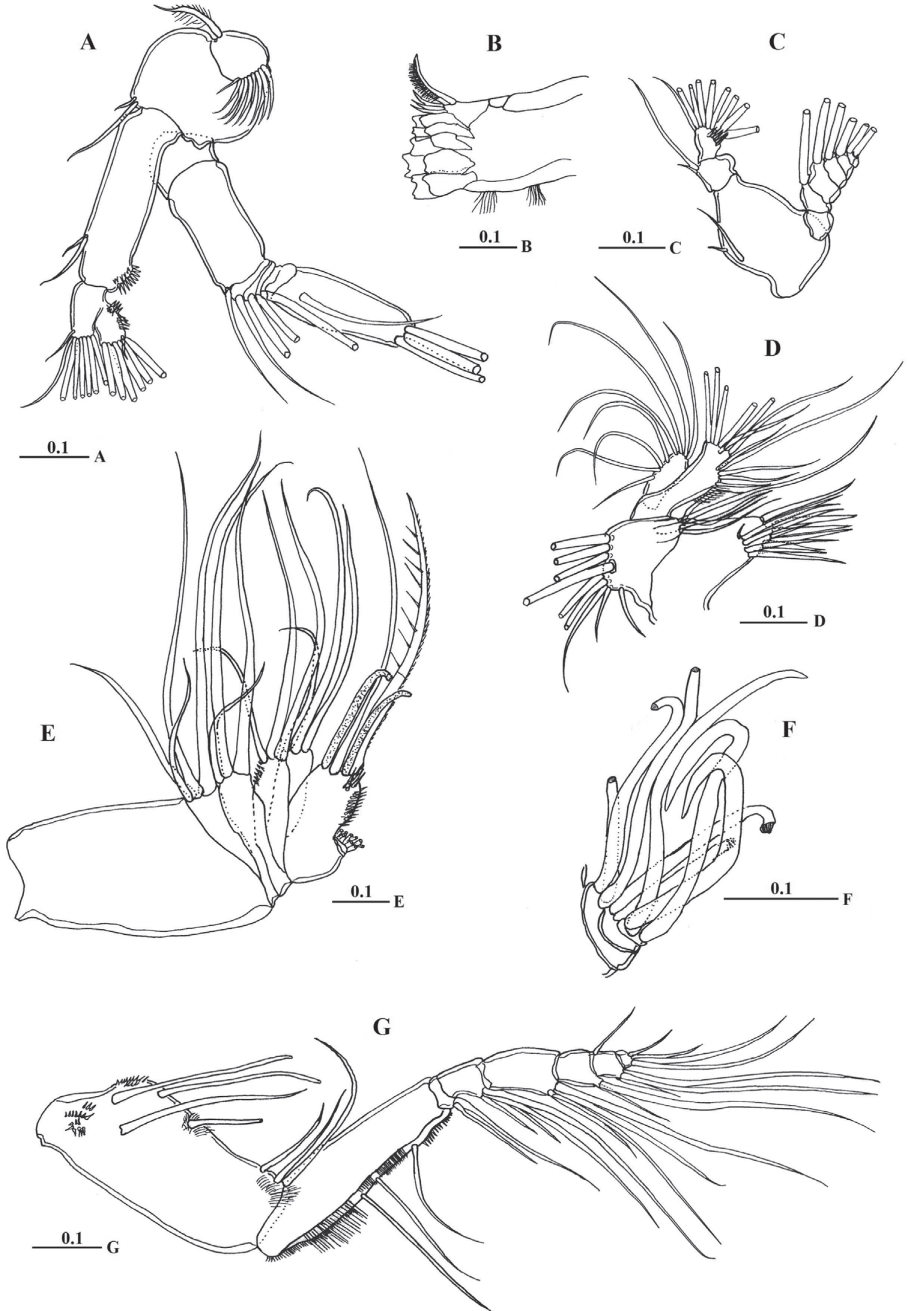
*Macandrewella cochinensis* female from the northern Red Sea. **A** antenna **B** mandibular gnathobase cutting edge **C** mandibular palp **D** maxillule **E** maxilla **F** maxilla endopod **G** maxilliped. All scale bars in mm.

Antenna (Fig. 4A) coxa with 1 plumose seta medially and lateral array of curved setules; basis with 2 mediodistal setae of unequal length. Exopod 7-segmented with setal formula of 0, 0-0-1, 1, 1, 1, 1, 1+3 setae; endopod 2-segmented, first segment with 2 subterminal setae and patch of fine setules medially, distal segment bearing 8 setae on middle lobe, terminal lobe with 7 setae and patch of fine setules.

Mandible gnathobase (Fig. 4B) heavily sclerotized with cutting edge bearing 8 teeth (5 of them flattened with broad edge) and spinulose seta. Palp (Fig. 4C) basis longer than wide, bearing 2 spinulose setae; exopod consisting of 5 segments with setal formula of 1, 1, 1, 1, 2; endopod 2-segmented, with 2 setae on first segment and 9 setae and row of fine spinules on second segment.

Maxillule (Fig. 4D) with praecoxal arthrite bearing 13 setae, 9 setae along terminal border, 4 setae on posterior surface and 1 seta on anterior surface (Fig. 4D). Coxal endite bearing 2 setae; coxal epipodite with 9 setae; basis completely fused with endopod; first and second basal endites with 3 and 5 setae respectively; baseoendopod with 7 setae terminally; exopod lobate, bearing 8 setae.

Maxilla (Figs 4E, F) praecoxal endite 1 with 4 setae, second praecoxal to second coxal endites each bearing 3 setae; basis with 2 setae and 2 worm-like sensory setae and patch of fine spinules. Endopod (Figs 3E, 4F) indistinctly three-segmented, bearing 3 brush-like, 2 brush-like and 3 worm-like sensory setae, respectively.

Maxilliped (Fig. 4G) praecoxal endites of syncoxa with 2 worm-like and 1 hirsute setae proximally, and 1 brush-like setae at nearly mid-length; coxal endite with 3 setae located at distal end. Basis nearly as long as syncoxa with submarginal row of minute spinules and 3 setae along medial margin. Endopod 6-segmented; first endopodal segment very short and almost incorporated into basis bearing 2 setae; second to sixth endopodal segment with setal formula of 4, 4, 3, 3+1, 4.

**Figure 5.**
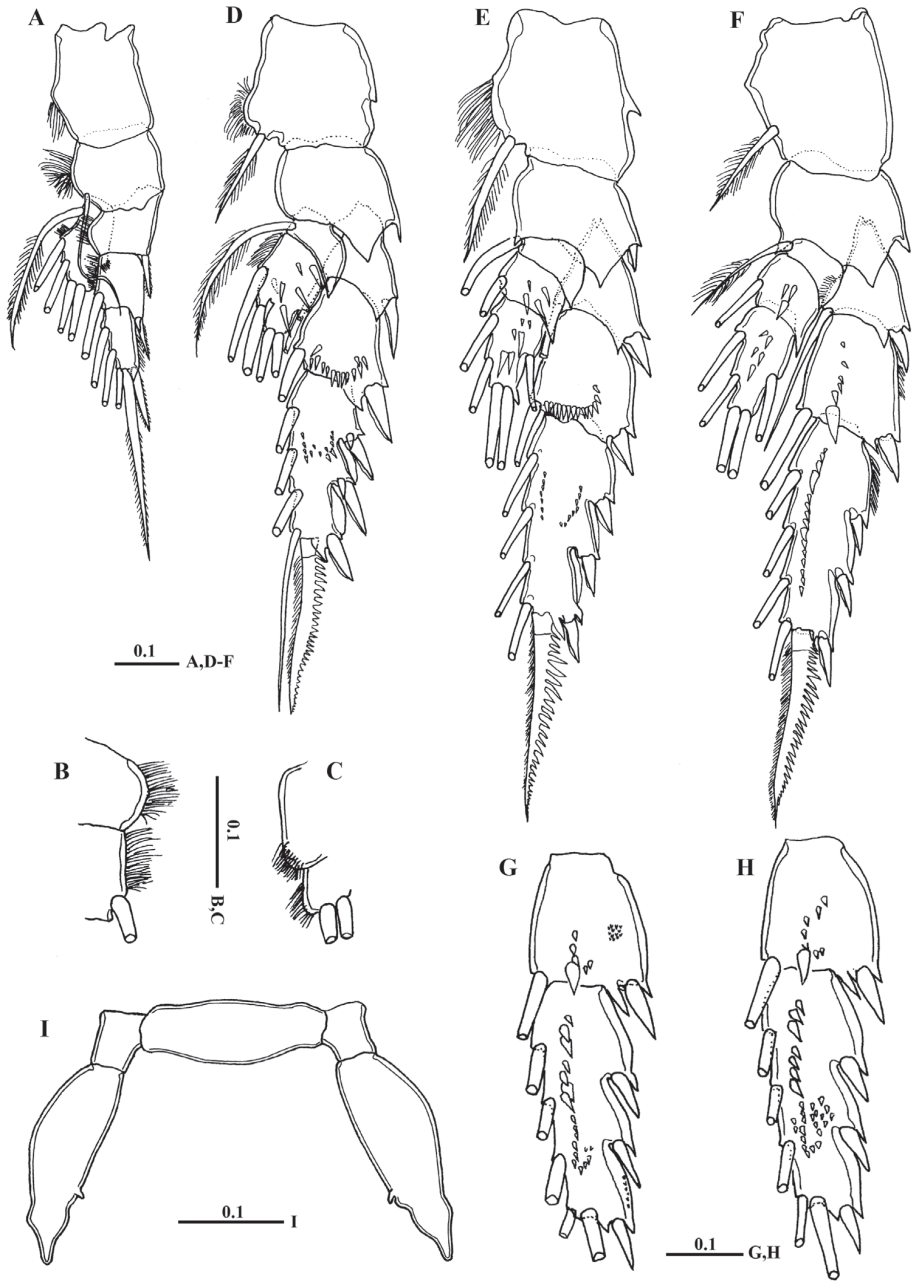
*Macandrewella cochinensis* female from the northern Red Sea. **A** Leg 1, anterior surface **B** medial margin of first and second exopodal segments of Leg 1 **C** lateral distal margin of leg 1 endopod **D** leg 2, posterior surface **E** Leg 3, posterior surface **F** leg 4, posterior surface **G–H** second and third exopodal segments of leg 4, anterior surface **I** leg 5, anterior surface. All scale bars in mm.

Legs 1 to 4 biramous, with 3-segmented exopods; endopod 1-segmented in leg 1, 2-segmented in leg 2, 3-segmented in legs 3 and 4. Spines and setal formula are shown in Table 1. Leg 1 (Figs 5A–C) smallest, first exopodal segment with expanded medial margin bordered by naked lateral spinules (Fig. 5B), middle segment bearing lateral spine and medial seta, distal exopod segment with serrate spine and spiniform terminal seta; endopod bearing middle lateral knob with patch of fine setules terminally (Fig. 5C). Leg 2 (Fig. 5D) coxa and basis with pointed prominence on lateral margin; second exopodal segment with crescent-like row of spinules on posterior surface; third segment with middle patch of spinules posteriorly; first endopodal segment without any spinules; second endopodal segment bearing 6 acute spinules. Leg 3 (Fig. 5E) coxa with pointed prominence on lamellar lateral margin; basis with pointed process on medial distal corner; second exopodal segment with crescent-like row of spinules along distal margin, third segment with minute spinules distributed in curved row; second and third endopodal segments bearing 4 and 6 spinules, respectively. Leg 4 (Fig. 5F): second and third exopodal segments each bearing longitudinal row of stout spinules distributed as shown in Fig. 5F. Shape, number and distribution of spinules along second and third exopodal segment varies among individuals (Figs 5G, H).

**Table 1.** Spines and setae formula of leg 1–4 of *Macandrewella cochinensis* collected from the northern Red Sea.

			Exopod			Endopod		
	Coxa	Basis	1	2	3	1	2	3
Leg 1	0-0	0-1	I-0;	I-1;	I,1,3	0,2,3		
Leg 2	0-1	0-0	I-1;	I-1;	III,I,4	0-1;	1,2,2	
Leg 3	0-1	0-0	I-1;	I-1;	III,I,4	0-1;	0-1;	1,2,2
Leg 4	0-1	0-0	I-1;	I-1;	III,I,4	0-1;	0-1;	1,2,2

Note: Roman numeral: spines; Arabic numeral: setae.

Leg 5 (Fig. 5I) rudimentary, 2-segmented separated at base; each terminal segment cylindrical with medial papilla-like protrusion and constriction at one-third distal part (see also Fig. 3F).

##### Male.

**Figure 6.**
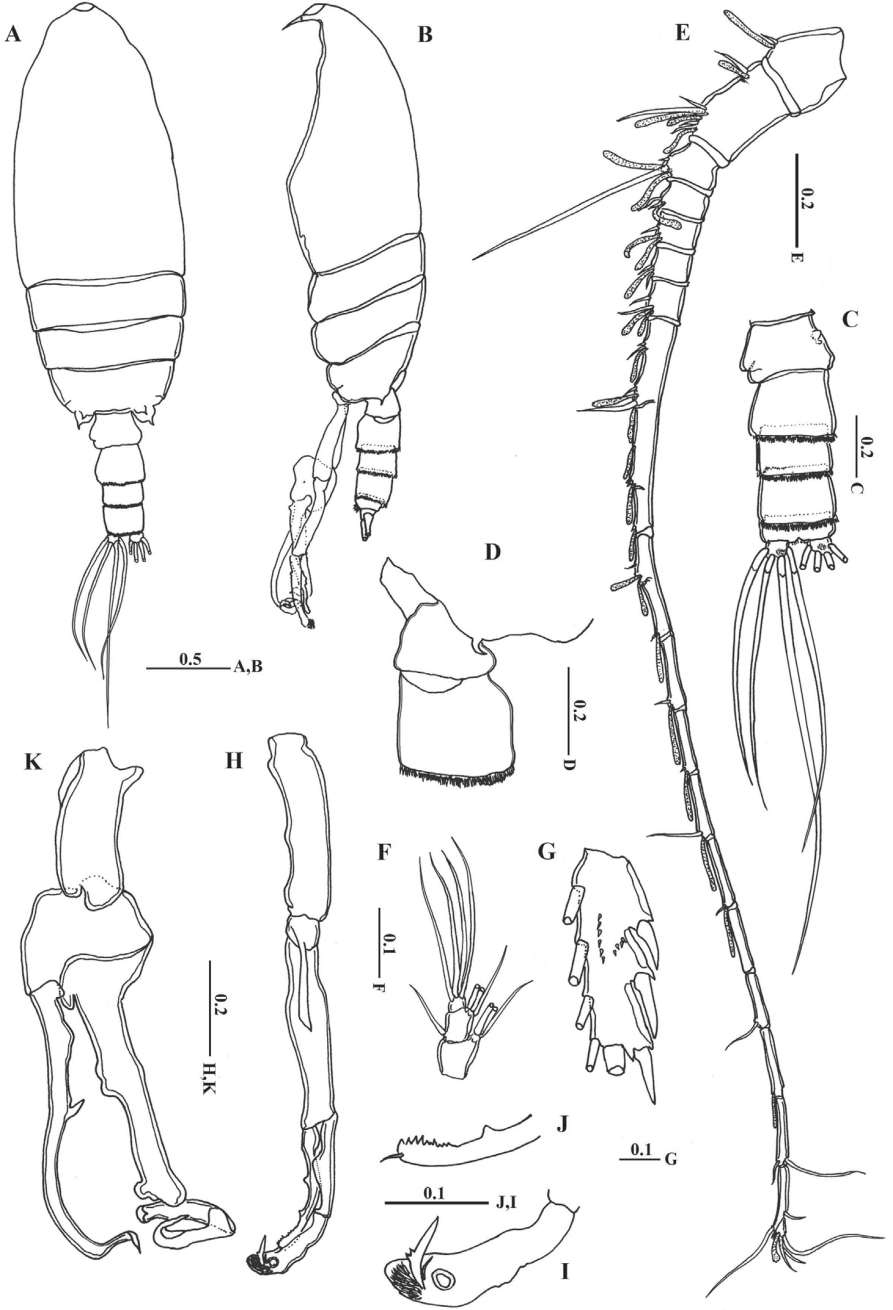
*Macandrewella cochinensis* male from the northern Red Sea. **A** habitus, dorsal view **B** habitus, lateral view **C** urosome, dorsal view **D** first and second urosomal segment, lateral view (right) **E** left antennule **F** maxilliped, terminal endopod segments **G** Exopod segment 3 of leg 2 **H** left leg 5 **I** terminal portion of left exopodal of leg 5 **J** terminal portion of left endopod of leg 5 **K** right leg 5. All scale bars in mm.

**Figure 7.**
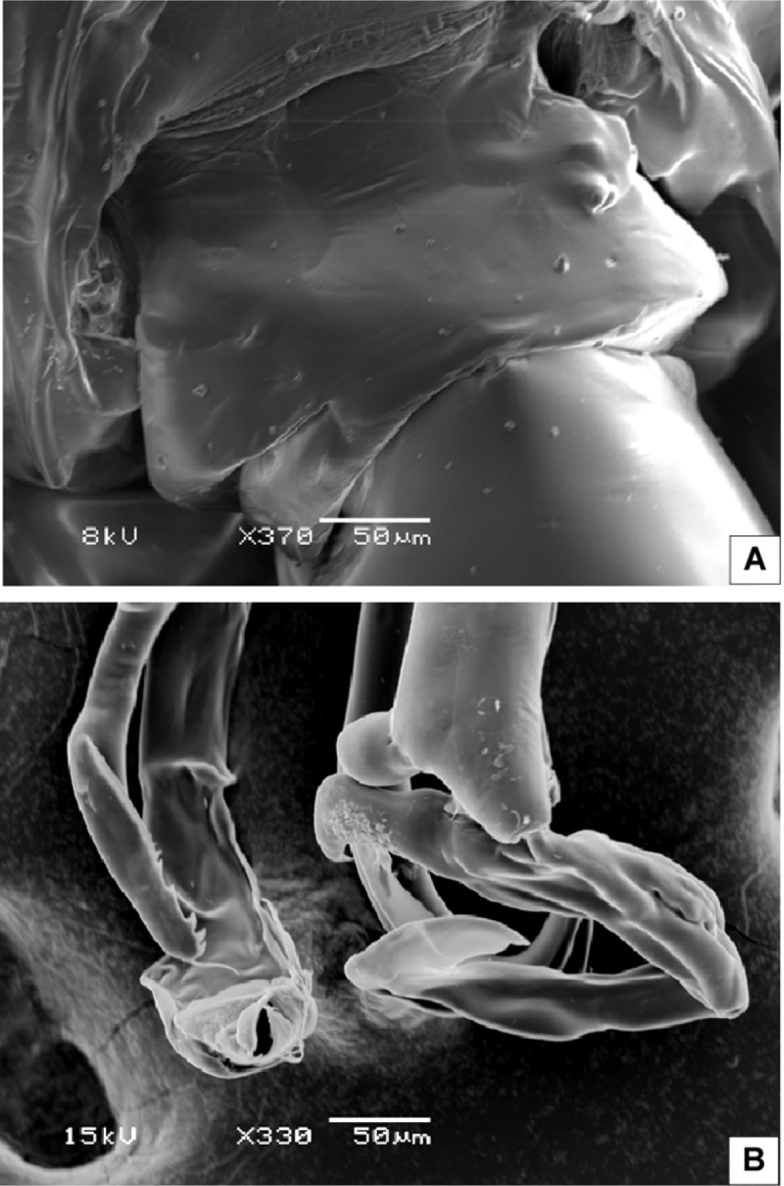
SEM micrographs of *Macandrewella cochinensis* male from the northern Red Sea. **A** genital somite, dorsal view **B** distal part of leg 5.

Body (Figs 6A, B) more slender than female; rostrum bifurcated with pair of filaments; cuticular median lens present at base of rostrum. Cephalosome completely fused with first pediger, fourth and fifth pedigers fused with suture visible laterally; border of fifth pediger symmetrical, ending with paired stout ventrally-curved processes. Urosome (Fig. 6C) 5-segmented; genital somite asymmetrical, with anterior dorsal knobs on right side (Figs 6C, D, 7A); second to fourth urosomites with thin spinules along posterior margin; second urosomite slightly asymmetrical in dorsal view, anal somite very small; caudal rami symmetrical, each ramus bearing 4 plumose setae.

Antennule (Fig. 5E) consisting of 18 and 19 articulated segments on right and left side, respectively. Setal formula of left antennule as follows: I-1+ae, II-IV-6+4ae (II-2+ae, III-2+2ae, IV-2+ae), V-2+2ae, VI-2+ae, VII-2 (1 missed)+2ae, VIII-2+ae, IX-2+2ae, X-XV-7+6ae, XVI-XVII-2+3ae, XVIII-1+ae, XIX-1+ae, XX-1+ae, XXI-1+ae, XXII-unarmed, XXIII-1, XXIV-1(missed)+ae, XXV-1+1, XXVI-1+1, XXVII-XXVIII-5+ae. Right antennules of 18 free segments with fusion of segments XXII and XXIII; setal formula of I-1+ae, II-IV-6+4ae, V-2+2ae, VI-2+ae, VII-1+ae, VIII-2+ae, IX-2+2ae, X-XV-5+6ae, XVI-XVII-2+3ae, XVIII-1+ae, XIX-1+ae, XX-1+ae, XXI-1+ae, XXII-XXIII-1, XXIV-1+1+ae, XXV-1+1+ae, XXVI-1+1, XXVII-XXVIII-5+ae.

Mouth parts and legs 1-4 similar to those of female except fifth and sixth endopodal segment of maxilliped with longer setae (Fig. 5F) and third exopodal segment of leg 2 with different number and distribution of posterior surface setules (Fig. 5G).

Leg 5(Figs 6H–K) elongated in general structure resembling that of the other species of the genus. Left leg (Fig. 6H) with coxa approximately as long as basis; basis with longitudinal keel–like structure along proximal half; exopod 2–segmented, second segment with lamellar plate covered with dense tuft of cilia and 2 elements terminally (Figs 6I, 7B); endopod one-segmented, shorter than exopod, bearing 2 medial triangular processes, one seta at tip and medially serrated margin (Fig. 6J). Right leg chelate (Fig. 6K); coxa with triangular expansion proximally; basis expanded laterally; first exopodal segment bearing 3 medial processes, one located proximally, middle irregular and distal somewhat triangular; second exopodal segment short, bearing internally directed process truncate curved at tip; third segment as long as previous segment, curved inward distally (Fig. 7B); endopod one-segmented, curved outward and recurved at tip, bearing round process distally and triangular process midway.

## Density and abundance of swarm

The density of *Macandrewella cochinensis* (adult and copepodites) in the studied area was about 422 individuals m^-3^. Adults constituted the major part of the swarm (72.5%), while copepodids consisted mainly of the fourth (CIV) and fifth (CV) stages, forming only 27.5%. Among adults, males show a slightly higher percentage in the population than females (38.1 and 34.4 % respectively). On the other hand, male copepodids (26.0%) outnumbered females (1.5%).

## Discussion

Original description of *Macandrewella cochinensis* by Gopalakrishnan (1973) is not enough detailed and contain only brief data on *Macandrewella* key characters valuable in congeners identification (Ohtsuka et al. 2002), e.g. projections of the female posterior prosomal borders, type of genital double-somite asymmetry, and female leg 5 present, or absent. In general, morphological characters of *Macandrewella* specimens collected from the northern Red Sea correspond to *Macandrewella cochinensis* and they are currently attributed to this species. However, their taxonomic status is expected to be proved when additional specimens from the *Macandrewella cochinensis* type locality will be obtained.

The studied specimens from the Red Sea differ from *Macandrewella cochinensis* sensu Gopalakrishnan (1973) in the following characters of females (features of Gopalakrishnan specimens are given in brackets): 1) pedigers 4 and 5 fused (separate); 2) pediger 5 with 2 pairs of processes, both dorsolateral projecting lamellar with serrated margin (apparently overlooked); 3) genital double-somite with asymmetrical anterodorsal protrusion on each side and posterodorsal swelling on left side (not described, only mentioned it is asymmetrical); 4) antennary first endopodal segment with 2 setae subterminally (1 seta); 5) endopodal middle lobe of distal segment with 8 setae (7 setae); 6) endopodal terminal lobe of distal segment bearing 7 setae (6 setae); 7) antennary second exopodal segment with long setae (seta shorter); 8) mandibular palp with basis carrying 2 setae (1 seta); 9) endopod segment 2 with 9 setae (10 setae); 10) maxillule praecoxal artherite with 13 setae (9 setae); 11) maxillule coxal epipodite bearing 9 setae (8 setae); 12) maxillule second basal endite with 5 setae (4 setae); 13) maxillule baseoendopod with 7 terminal setae (6 setae); 14) maxilliped endopod with setal formula of 2, 4, 4, 3, 3+1, 4 (vs 2, 4, 4, 3, 2+1, 4); 15) leg 3 basis with lateral prominence (absent); 16) surface spinulation of swimming legs is more dense than in the original description; 17) leg 5 rudimentary, composed of 2 segments (apparently overlooked). Males from the Red Sea and described by Gopalakrishnan (1973): differ in second exopodal segment of left leg 5 with 1 seta and 1 element terminally (1 element).

*Macandrewella cochinensis* closely resembles *Macandrewella stygiana* Ohtsuka, Nishida & Nakaguchi 2002 in dorsolateral processes on the prosomal ends of the female serrated and in the left ventrolateral process of the prosomal border extending nearly posterior margin of the genital double-somite. However, *Macandrewella cochinensis* is readily distinguishable from *Macandrewella stygiana* in the following characteristics: 1) the second and third urosomites are nearly equal in length (second urosomite longer than third one in *Macandrewella stygiana*); 2) female caudal left seta V 1.5 times longer than right (more than 2 times longer in *Macandrewella stygiana*); 3) female leg 5 is cylindrical, composed of 2 segments with 1 medial process and a constriction on the distal third of the distal segment (more flattened in *Macandrewella stygiana*); 4) the lateral middle process of the right endopod of male leg 5 is larger (smaller); 5) the lack of a medial distal process of the second exopodal segment of male right leg 5 (present); 6) the distal exopodal segment of male right leg 5 is relatively narrower in *Macandrewella cochinensis* (broader in *Macandrewella stygiana*).

The female of *Macandrewella cochinensis* is also similar to that of *Macandrewella joanae* Scott 1909 collected from Halmahera Sea, Indonesian Archipelago, but can be distinguished by the presence of ventrolateral processes on the distal prosomal borders that reach nearly to the midlength of the genital double-somite; the genital operculum is wider than long; the left middle seta on the caudal ramus is nearly 1.5 times as long as the right one but shorter than in *Macandrewella joanae*; the terminal segment of the female leg 5 is more reduced than in *Macandrewella joanae* and has no terminal elements.

Swarm formation is known in coastal and deep-sea calanoid families such as Acartiidae, Calanidae, Centropagidae, Pontellidae, Pseudodiaptomidae, Ridgewayiidae, Spinocalanidae, Temoridae, and Tortanidae (e.g. Hamner and Carleton 1979, Fleminger 1983, Ueda et al. 1983, Mauchline 1998, Heidelberg et al. 2010). However these species except for the Spinocalanidae form multispecies assemblages (Fleminger 1983, Mauchline 1998, Ivanenko et al. 2007). This is the first record of the family Scolecitrichidae to form a monospecific aggregation. The adaptive meaning of copepods’ swarming is interpreted as being possibly related to: (1) antipredation against visual predators; (2) reduction of dispersion by currents; (3) facilitating and enhancing mating opportunity; (4) keeping position to feed on coral mucus (Mauchline 1998); (5) positioning in the volcanic gases (Fleminger 1983, Ivanenko et al. 2007). In case of *Macandrewella cochinensis*, the dominance of adult and swarming position near the surface are peculiar, suggesting the likelihood of the above-mentioned first and third possibilities. Many studies showed that most members of the family Scolectrichidae are detritivores (e.g. Nishida et al. 1991, Nishida and Ohtsuka 1997). Regarding *Macandrewella*, Ohtsuka et al. (2002) in their study concluded that this genus is omnivorous voraciously feeding mostly on small crustacean carcasses and/or sloughs as well as radiolarians and diatoms.

## Supplementary Material

XML Treatment for Macandrewella cochinensis
